# Does the whitening dentifrice containing activated charcoal interfere with the properties of dental enamel? Microhardness, surface roughness and colorimetry analyzes

**DOI:** 10.4317/jced.60785

**Published:** 2024-03-01

**Authors:** Gabriela-Conde Santos, Juliana C. P. Baia, Mara E.S. Ribeiro, Taynara N. B. Silva, Mário H. Silva e Souza Junior, Sandro C. Loretto

**Affiliations:** 1 Department of Restorative Dentistry, UFPA-Federal University of Pará, Belém 66075-110, Brazil

## Abstract

**Background:**

The study evaluated the influence of whitening dentifrice containing activated charcoal on microhardness (MH), surface roughness (Ra) and colorimetry of tooth enamel.

**Material and Methods:**

A total of 60 healthy bovine incisor teeth were used and divided into: G1 (regular non-whitening toothpaste), G2 (conventional whitening toothpaste), G3 (activated charcoal based whitening toothpaste) and G4 (10% carbamide peroxide gel - PC10). Groups G1, G2 and G3 underwent simulated toothbrushing for 14 days, while G4 received bleaching treatment for the same time. The readings of MH, Ra and colorimetry were performed before any intervention (T0 - baseline) and at the end of 14 days of the proposed treatments (T1). After confirming the normality of the data, the results of MH, Ra and colorimetry were subjected to 2-factor ANOVA for repeated measures (α = 5%).

**Results:**

For MH, there were no statistical differences in G1, G2 and G3, only in G4. Considering Ra, a significant increase was observed in G2 and G3, with no statistical differences found in the other groups. Regarding colorimetry, the average color difference (ΔE) was greater in the G4 group (11.30 ± 4.31), even compared to the groups submitted to the whitening dentifrices: G2 (5.13 ± 2.75) and G3 (5.86 ± 3.66).

**Conclusions:**

It was concluded that the use of a whitening toothpaste containing activated charcoal caused deleterious effects on the enamel Ra, but did not affect the microhardness of the substrate, besides promoting a color change inferior to the regular non-bleaching toothpaste or PC10 gel.

** Key words:**Whitening dentifrice, charcoal, roughness, microhardness.

## Introduction

Tooth color change is one of the most common complaints reported by patients who seek dental offices for aesthetic treatments (1). Therefore, tooth whitening has become quite popular, increasing its demand in recent decades, as it provides fast and expressive results, without wearing down the tooth structure (2). However, this practice can cause side effects, such as morphological changes in the microtopography (3), microhardness and surface roughness of the enamel, in addition to transient sensitivity (4).

An alternative to mitigate, or even avoid these risks, has been made available by the dental industry through the different compositions of whitening toothpastes, which are considered a category of simpler and less expensive homemade products for those who wish to have whiter teeth, guaranteeing results within 2 to 4 weeks of continuous use (5,6). Some of these toothpastes have low concentrations of hydrogen peroxide in their formulation, while others contain abrasive components capable of promoting the removal of extrinsic stains (6,7). Studies have focused on the effects of these whitening dentifrices on the integrity of the dental surface, with some reporting the presence of morphological and hardness alterations (1,8), which raises disagreements about the effects that the uninterrupted use of these products may cause (8).

Although the terms “tooth bleaching” and “tooth whitening” are frequently used, both in the literature and in clinical practice, they are technically not the same. Bleaching is a process involving an oxidative chemical that alters the light-absorbing or light-reflecting nature of a material structure, increasing its perception of whiteness. In contrast, tooth whitening is achieved using low concentration peroxides and/or mechanical approaches to remove surface stains with the use of professionally applied abrasives through prophylactic pastes, and also through tooth brushing with a whitening toothpaste (9). These commercially available toothpastes have “whitening” properties, as they whiten teeth to different degrees by removing superficial (extrinsic) stains, which can be removed relatively quickly, for example, in smoking patients (8).

More recently, whitening toothpastes containing activated charcoal have been widely publicized and accepted by society, as they have the ability to adsorb pigments (chromophores) responsible for changing the color of teeth (1). The active ingredient contained in commercial toothpastes is a fine form of powdered activated carbon, oxidized by controlled reheating or by chemical means, and can be obtained from a variety of carbon-rich materials. Coal in fine powder is a material with variable abrasiveness, depending on the source and methods used to prepare and grind this coal (1,10).

However, the use of toothpastes based on this compound (charcoal) deserves attention, as conventional whitening toothpastes already have a considerable rate of abrasiveness (10). Similar to the abrasion caused by whitening toothpastes, activated charcoal can achieve a whiter appearance by removing surface stains and plaque due to their abrasive action. But usually, these products do not change the intrinsic color of the tooth, which is largely determined by the color of dentin (8,11,12). However, it has been reported that activated charcoal is more abrasive than other whitening toothpastes and is not suitable for intraoral use (13,14). In addition, the incorrect use associated with other vehicles with the aim of whitening may increases the surface roughness of the enamel (15).

Palandi *et al*. point out that the incorporation of this mineral can lead to damage to the ultrastructure of dental enamel, affecting its hardness and altering the surface of the substrate, making it more porous (16). Nonetheless, Franco *et al*. reported no substantial difference for surface roughness in bovine enamel mechanically brushed either by conventional (1450-ppm F) toothpaste or activated charcoal-based tooth powder after 14 days (17). From a practical point of view, it is important to know the whitening performance of these toothpastes (containing activated carbon), evaluating them through more reliable instrumental methods, such as spectrophotometers and colorimeters (18), in order to objectively prove their effectiveness.

Therefore, the objective of this study was to evaluate the influence of a whitening toothpaste containing activated charcoal on dental enamel through microhardness, surface roughness and colorimetry analyzes.

## Material and Methods

-Obtaining, characterization of the sample and ethical aspects

The sample calculation necessary to undertake statistical inference was performed at a correlation of 0.5, with a power of 80% and a significance level of 5%, using the BioEstat® software (Civil Society Mamirauá). This research was approved by the Ethics Committee on the Use of Animals (CEUA) under protocol n° 4273240320.

Sixty bovine incisor teeth (species *Bos taurus indicus*, with an average age of 24 months) were used, obtained from animals slaughtered at the Cooperativa da Indústria Agropecuária do Pará (SOCIPE, Belém, Pará, Brazil). The teeth were erupted in the oral cavity and had a sound crown and complete root formation. After the extractions, the teeth were disinfected (0.1% thymol - A Formula, Belém, PA, Brazil) for 1 week. Then, they were washed in running water, any traces of blood or adhered tissue removed, and analyzed using a stereoscopic magnifying glass (SZ2-ILST, Olympus SZ61, Tokyo, Japan) (40x) to assess their structural integrity (elements that had cracks or fractures were discarded).

The dental crowns were submitted to two cross sections using a double-sided diamond disc (KG Sorensen, Cotia, SP, Brazil). The first cut was made at a distance of 15 mm from the cementoenamel junction, measured with a digital caliper (DIN 862; Mitutoyo, São Paulo, SP, Brazil), and parallel to the incisal edge. The second one was performed 5 mm from the cementoenamel junction, thus obtaining samples of the middle portion of the dental crown with a height of 10 mm. The dental fragments had the buccal surface inserted in wax and were embedded in chemically activated acrylic resin (JET, Clássico, Campo Limpo Paulista, SP, Brazil), using acrylic matrices with a height of 11 mm. Twenty-four hours after the inclusion of the specimens, the samples were flattened in a semi-automatic polisher (Aropol VV-PUD, Arotec, Cotia, SP, Brazil) with constant cooling, at low speed (200rpm), under a force of 20N, using silicon carbide sandpaper in decreasing order of granulation (#600, #800, #1200 and #2000), for 30s each. Every change of a sandpaper, specimens were washed for 3min in an ultrasonic bath (TD30 Plus; Bio -Art, São Paulo, Brazil), so that there was no interference from the grains of the sandpaper previously used.

**Figure 1:**
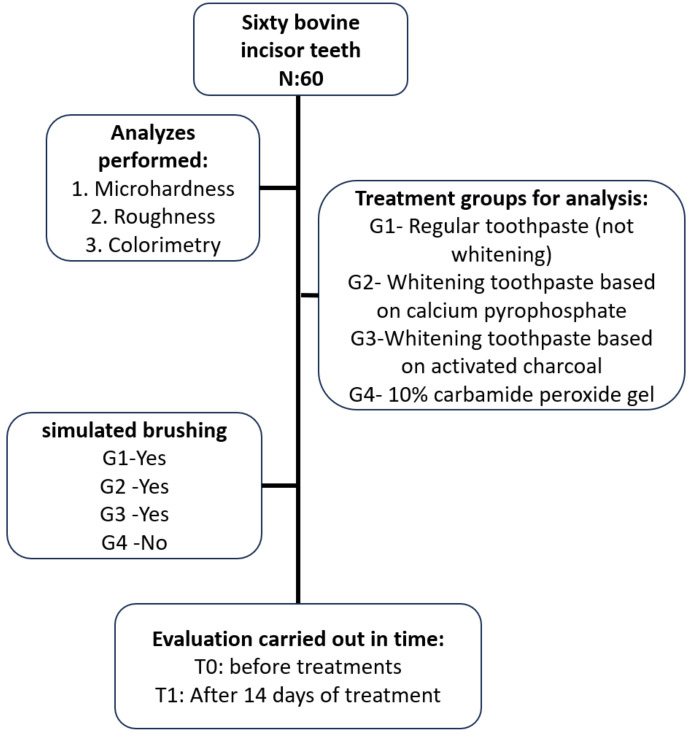
Flowchart with summary of the methodology of the groups with the analyses performed.

After, specimens were randomly distributed (through the BioEstat® software - Civil Society Mamirauá) in the research groups as described in Figure 01 (n=15), and the initial readings were performed. After performing the initial measurements of colorimetry, surface roughness and microhardness, the specimens were subjected to simulated toothbrushing or tooth bleaching. Materials used in experimental groups, and their respective compositions, are shown in Table 1, (Fig. 1).

**Table 1:**
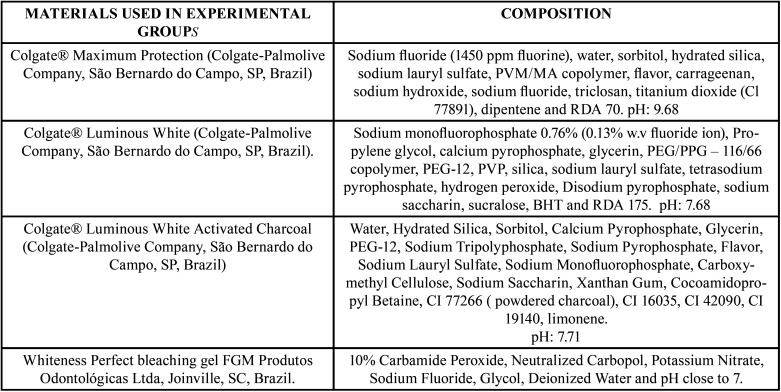
Division of experimental groups regarding the intervention to be performed.

-Microhardness analysis

For each experimental group, readings were performed before any intervention (baseline), and after 14 days of the experimental interventions: simulated toothbrushing for groups G1, G2 and G3, and tooth bleaching with PC10 for group G4. Knoop microhardness (KHN) was measured by a microhardness tester (HMV-2; Shimadzu, Kyoto, Japan), with a static load of 0.49 N (50 g) applied for 20 seconds (19), where the 3 indentations, spaced by 100 µm, were carried out. The microhardness value of the specimen was determined by the arithmetic mean of the 3 readings. The enamel area of the specimens was divided into two halves, so that the microhardness and roughness measurements were carried out in different areas. Besides, the area designated for the microhardness readings, was additionally subdivided into 2 parts, so that there was no coincidence of the already indented areas.

-Surface roughness analysis

For each experimental group, surface roughness readings were taken before (baseline) and after any experimental interventions. A roughness meter (SJ-301; Mitutoyo, Los Angeles, CA, USA) was used, where the parameter adopted to obtain the surface roughness was the arithmetic roughness (Ra), determined by the average of 3 readings, with a trace limit (Lt ) of 5mm, and with a sampling length or cut-off (La) of 0.25mm (4).

-Colorimetric analysis

**Figure 2:**
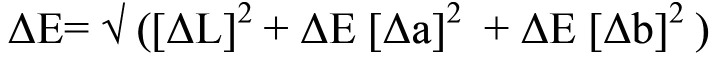
Formula.

Colorimetric readings were taken for each experimental group before (baseline) and after any experimental interventions, using a VITA Easyshade Compact spectrophotometer (VITA Zahnfabrik, Bad Säckingen, Germany), based on the CIE L * a * b * system. To obtain standardized color measurements, the pointer of the device and surfaces of the specimens were established parallelly to each other and three measurements were obtained for each specimen and averaged. To assess the color change between the initial and final results, ΔE was calculated using the following formula: (Fig. 2).

-Simulated dental brushing

For the specimens that received simulated toothbrushing - conventional toothpaste, whitening toothpaste based on calcium pyrophosphate, or whitening toothpaste based on activated charcoal, an Oral B Professional Care 500 electric toothbrush (Oral B, Schwalbacham Taunus, Germany), fixed on an appropriate support, was used in “continuous mode”. Specimens were accommodated in customized brushing cocoons, allowing efficient and uniform performance of the brush bristles, having a “stop” to accommodate the final brush base, stabilizing it during use. Each surface of the specimen received a daily cycle of 15 seconds (20) each, for 14 days (16), with a load of approximately 200g (2N) on the brushes. For brushing, a dilution of toothpaste and distilled water (1:3, weight-volume ratio) (21) was prepared immediately before use, in order to preserve its characteristics, and was deposited on the surface of the specimen. At the end of brushing, the specimens were removed, washed for 30 seconds with distilled water and dipped in artificial saliva, followed by storage in a biological oven (37°C/24h).

-Tooth bleaching

For the group that received the bleaching treatment, Whiteness Perfect 10% carbamide peroxide (PC10) (FGM, Joinville, SC, Brazil) gel was used, applied daily for 4 hours, for 14 days (manufacturer’s instructions), with a proportion of 0.1ml of bleaching gel to 0.05ml of artificial saliva (4), dispensed on acetate trays previously made for each specimen, in order to facilitate the application of the product, and thus trying to reproduce the conditions of home bleaching. During the 4-hour period, the specimens were placed in a plastic container with a water blade at the bottom to maintain 100% humidity and placed in a biological oven (37°C). After daily application, the specimens were washed with an air/distilled water spray (1 min), at a distance of approximately 5 cm from the enamel surface exposed to the intervention. Then, the specimens were immersed in artificial saliva and taken to the biological oven (37°C/24h).

-Statistical analysis

Statistical analysis was performed using the BioEstat® software (Sociedade Civil Mamirauá). To confirm the normal distribution of data, the Shapiro-Wilk test was used. Intragroup statistical analysis for microhardness and roughness data was performed using Student’s t test for related samples. For the color analysis, the one-way ANOVA test with Tukey’s test was performed. All statistical analyzes adopted a significance level of 5% (*p*≥0.05).

## Results

The Table 2 shows the means (standard deviation) of the microhardness values. In the G4 group at T1 (after interventions), the lowest average in the microhardness values was observed (221.19), with the highest one found in the same group at T0 (319.06). No intragroup statistical difference was observed in G1, G2 and G3, and only the G4 group showed a significant reduction in microhardness after 14 days of bleaching, (Table 2).

**Table 2:**
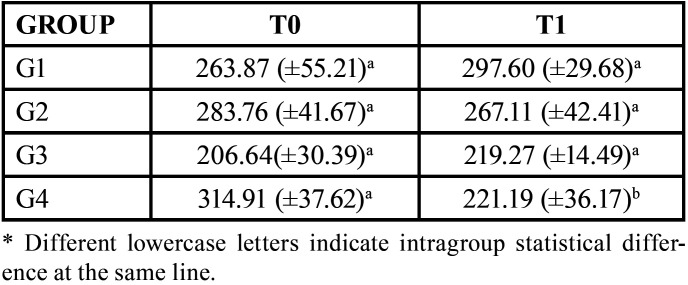
Means (standard deviations) of microhardness according to treatment time for the groups tested.

The Table 3 shows the averages (standard deviation) of the surface roughness values. The highest roughness mean was found in the G2 group at T1, and the lowest one at T0 in the same group. An increase in roughness was observed only in groups G2 and G3, which showed a statistical difference in the intragroup comparison.

The Table 4 shows the colorimetry means and standard deviations of ΔE for each experimental group. The mean ΔE was higher in the G4 (11.30 ±4.31) than in the other groups, including those submitted to the abrasive challenge with whitening toothpastes: G2 (5.13 ±2.75) and G3 (5.86 ±3.66).

**Table 3:**
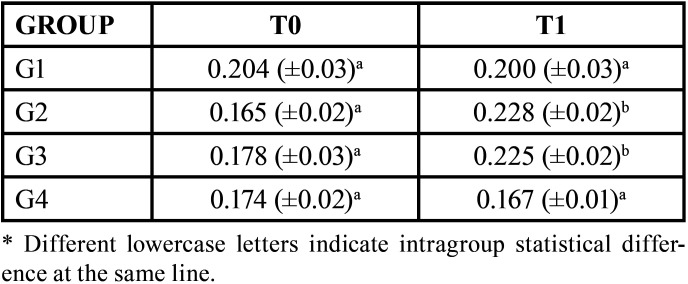
Means (standard deviations) of surface roughness according to treatment time for the groups tested.

**Table 4:**
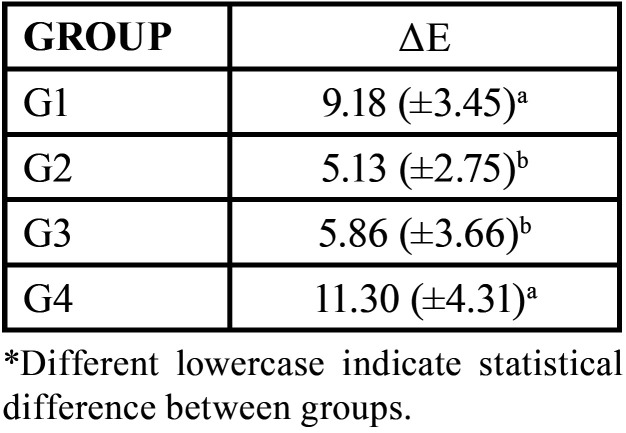
Means (standard deviations) of the ΔE color difference, according to the treatment time for the groups tested.

## Discussion

The use of whitening toothpastes containing activated charcoal to obtain whiter teeth has become quite popular (22). However, it is still necessary to be cautious when using these toothpastes, as their indiscriminate use can affect the enamel surface, according to the results of the present study. In this sense, it was demonstrated how much whitening dentifrices can affect the surface roughness of the enamel after daily use for 14 days. Besides, the toothpaste containing activated charcoal lead to no significant alteration on enamel microhardness, but was as effective as the other whitening dentifrice tested on enamel color changing. However, it is important to emphasize that both whitening toothpastes promoted lesser enamel color change than bleaching gel (10% carbamide peroxide).

Bleaching with a 10% carbamide peroxide gel is considered a safe and effective treatment, especially if performed under professional supervision, but it can cause adverse effects on the tooth structure, such as changes in enamel microhardness (23). Preserving the microhardness of the substrate is essential for the maintenance of oral health and integrity of the dental structure, as it reflects on the ability of the tooth to resist masticatory forces, mechanical and chemical challenges (24).

Some studies have been performed to assess possible damage to dental substrates after bleaching treatments (25,26), and have demonstrated that enamel microhardness can be altered after exposure to PC10 under a variety of experimental conditions (27,28). The oxidizing agents present in bleaching gels they are capable of modifying the chemical composition of the enamel, reducing the concentrations of important components such as calcium, phosphate and fluorine (28,29). The reduction in enamel microhardness detected after bleaching may be related to mineral loss, which modifies the morphology of the surface prisms of the substrate (30). This justifies the reduction in the microhardness values of the G4 group, which underwent treatment with the bleaching agent PC10, while the groups that underwent simulated toothbrushing did not show significant differences after 14 days of treatment.

It has been shown that toothpastes with a pH below the critical one for demineralization of the tooth structure can promote a greater abrasive action by a possible association of erosive and abrasive effects (31). In the present study, the toothpastes had a neutral pH or above 7, which may explain the microhardness values of groups G1, G2 and G3 (where no significant differences were observed).

Regarding the composition, all toothpastes used in the present study contained fluoride. One study evaluated whitening toothpastes with and without fluoride and observed that the latter (without fluoride) exhibited greater mineral loss, while fluoride products had the expected cariostatic effect as a result of the remineralization process (32). Furthermore, it has already been shown that brushing with a fluoride toothpaste helps to prevent the decrease in surface microhardness during the bleaching treatment, while the absence of this element results in less microhardness (33).

The surface roughness results showed a significant increase in G2 and G3, both groups that used whitening toothpastes. The literature states that these products (whitening toothpastes) are capable of affecting the surface roughness of enamel (34). Therefore, the excessive use of more abrasive toothpastes, combined with more vigorous brushing, not only works to remove stains, but can also promote excessive enamel wear (35). In our study, the activated charcoal toothpaste group (G3) provided an increase in roughness as well as the calcium pyrophosphate-based whitening toothpaste (without activated carbon) (G2), which raises concern, since associating an ingredient such as activated charcoal acting as an abrasive in the whitening toothpaste compositions would further affect the roughness of the substrate. It has been proven that groups treated with powdered charcoal cause an increase in enamel roughness, due to the abrasive effect of charcoal on this surface (16,36).

In the present study, the G4 group did not present significant roughness alterations. However, the literature states that the use of whitening toothpastes associated with whitening with PC10 are able to increase the roughness of the substrate (35), which was not performed in the present study (G4 was not exposed to an abrasive challenge – simulated toothbrushing), indicating that the bleaching therapy seems to not affect the roughness of the substrate, only the microhardness.

Different *in vitro* (36,37) and clinical (38,39) studies have already investigated the effectiveness of color change promoted by whitening dentifrices, and the literature still shows contradictory results. Pintado *et al*. did not observe enamel color changes with the use of whitening dentifrices during a four-week evaluation period (6). In the present study, the group responsible for the greatest color change was the G4 group, which underwent bleaching therapy, followed by the control group (regular non-whitening toothpaste), and finally the groups that used whitening toothpastes (with and without activated charcoal). The abrasive agents present in whitening toothpastes are capable of removing extrinsic stains, which should not be confused with a real bleaching effect (40). Therefore, these whitening toothpastes are often considered just surface stain removers (6).

CIELab color system coordinates (L *, a *, b *) locate the object in a 3D color space and quantify the differences in brightness and chromaticity (ΔL*, Δa*, Δb*), for the color difference total (ΔE) (40). In this study, the ΔE difference of the G4 group is highlighted, promoting a noticeable lightening, but there is also a color change in the G1 control group, emphasizing that both groups (G1 and G4) did not present an increase in roughness. There is a direct relationship between color and roughness, due to the texture of the tooth surface, associated with the light reflectance of the enamel (18,41). Rougher surfaces modify the absorption and allow diffuse light scattering, unlike smoother surfaces, which have more specular light scattering (42). This justifies the ΔE results of the G1 group being higher than those of G2 and G3, both represented by whitening dentifrices.

For now, the literature is still unsure about the exact effects of whitening dentifrices containing activated charcoal on tooth color. Vaz *et al*. evaluated toothpastes containing activated charcoal, and demonstrated superior *in vitro* whitening efficacy for the ones with abrasive particles and containing the blue covarine pigment, than for those containing activated charcoal (43). Similarly, other studies tested the performance of charcoal powder and reported no effectiveness on tooth whitening (17,44). On the other hand, *in vitro* studies by Palandi *et al*. and Ghajari *et al*. showed evidence of the effect of charcoal, the former detecting minor whitening effects (16), and the latter through and *in vitro* study with human enamel, revealing significant changes in the color of the teeth after toothbrushing (13). Therefore, it is still necessary to investigate the possible adverse effects caused by whitening toothpastes containing activated charcoal, in addition to aspects not yet investigated, such as dental sensitivity, for example. The literature requires more evidence, mainly clinical, on the safety and efficacy of these products.

## Conclusions

Within the limitations of this study, it was concluded that the use of whitening toothpaste containing activated charcoal significantly increased the surface roughness of the enamel but did not influence its microhardness. In addition, this dentifrice promoted a less notable color change than the bleaching gel based on 10% carbamide peroxide or regular non-whitening toothpaste.
